# Botulinum Toxin-Chitosan Nanoparticles Prevent Arrhythmia in Experimental Rat Models

**DOI:** 10.3390/md18080410

**Published:** 2020-08-02

**Authors:** David Sergeevichev, Vladislav Fomenko, Artem Strelnikov, Anna Dokuchaeva, Maria Vasilieva, Elena Chepeleva, Yanina Rusakova, Sergey Artemenko, Alexander Romanov, Nariman Salakhutdinov, Alexander Chernyavskiy

**Affiliations:** E. Meshalkin National Medical Research Center of the Ministry of Health of the Russian Federation, 15 Rechkunovskaya Str., 630055 Novosibirsk, Russia; vladislav@ngs.ru (V.F.); agstrelnikov@gmail.com (A.S.); a_dokuchaeva@meshalkin.ru (A.D.); vasilievam@yandex.ru (M.V.); e_chepeleva@meshalkin.ru (E.C.); yarojana@mail.ru (Y.R.); s_artemenko@meshalkin.ru (S.A.); abromanov@mail.ru (A.R.); anvar@nioch.nsc.ru (N.S.); amchern@mail.ru (A.C.)

**Keywords:** botulinum toxin A1, chitosan nanoparticles, antiarrhythmics, pharmacological models of arrhythmia, electrically induced arrhythmia

## Abstract

Several experimental studies have recently demonstrated that temporary autonomic block using botulinum toxin (BoNT/A1) might be a novel option for the treatment of atrial fibrillation. However, the assessment of antiarrhythmic properties of BoNT has so far been limited, relying exclusively on vagal stimulation and rapid atrial pacing models. The present study examined the antiarrhythmic effect of specially formulated BoNT/A1-chitosan nanoparticles (BTN) in calcium chloride-, barium chloride- and electrically induced arrhythmia rat models. BTN enhanced the effect of BoNT/A1. Subepicardial injection of BTN resulted in a significant antiarrhythmic effect in investigated rat models. BTN formulation antagonizes arrhythmia induced by the activation of Ca, K and Na channels.

## 1. Introduction

Botulinum toxin (BoNT) is a safe and efficient therapeutic means to treat a variety of conditions characterized by the hyperfunction of nerve terminals [1,2]. Recently, there has been a growing interest in BoNT for the treatment of atrial fibrillation (AF). Initially, Tsuboi et al. [3] demonstrated that BoNT injected into the sinoatrial fat pad inhibited a decrease in sinus rate in response to vagus nerve stimulation and suggested that BoNT can inhibit ganglionic neurotransmission in the dog heart in situ. Later, Oh et al. [4] demonstrated that direct injection of BoNT in epicardial fat pads temporally suppressed AF inducibility in dogs. In the first clinical study of BoNT effects on patients undergoing coronary artery bypass surgery, Pokushalov et al. [5] demonstrated that BoNT injection suppresses postoperative atrial fibrillation. Recently, Lo et al. [6] demonstrated that suppression of the four major atrial ganglionated plexi by BoNT may break the vicious cycle of “AF begets AF” by inhibiting autonomic remodeling, and possibly preventing subsequent progression of AF to more persistent forms. In addition, Nazeri et al. [7] found that after one week following injection of BoNT into the atrial fat pads of sheep, the vulnerability of atrial tissue to AF induction and the vagal influence on the atrial effective refractory period were reduced compared to baseline levels.

However, the assessment of antiarrhythmic properties of BoNT has only been studied using experimental models of vagal stimulation [3,4,7] and rapid atrial pacing [6]. Indeed, although the effect of BoNT on atrial arrhythmias has attracted much attention, the effects of BoNT on ventricular arrhythmias remain unknown.

Therapeutic doses of botulinum neurotoxin drugs are safe, and side effects are relatively rare. Adverse effects often depend on the injection site: there are skin rash, muscle weakness, fatigue, flu-like symptoms, a dry mouth and dizziness [8]. However, in the “first-in-human” study of the epicardial fat pad botulinum toxin injection for atrial fibrillation prevention, there was no serious adverse effect during the one-year and three-year follow-up period [5,9].

The BoNT block of neuromuscular transmission occurs after a lapse of time [7,10,11] and the blocking effect is temporary, with recovery of neuromuscular transmission within one to six months in skeletal muscles [12] and within three weeks in the heart [4]. Therefore, new formulations that may accelerate the effect of BoNT and increase its duration time are highly desirable. Recently, we have demonstrated that globular chitosan prolongs the block of neuromuscular transmission after the BoNT intramuscular injection in rats [13]. Chitosan is a linear polymer derived from chitin, the second most abundant aminopolysaccharide after cellulose. It is fully biocompatible, studied in numerous pharmaceutical and medical applications, demonstrating the highest possible safety profile [14]. To overcome the poor solubility of linear chitosan in water, we used an improved globular chitosan, Novochizol. Novochizol synthesis comprises a two-step activation of linear chitosan, an intramolecular reaction that cross-links linear chitosan molecules. After the cross-linking procedure, Novochizol can be impregnated with active pharmaceutical ingredients [15].

Therefore, in the present study, we examined the antiarrhythmic effect of BoNT and its formulation with an enhanced globular chitosan (Botulinum_Novochizol, BTN) using calcium chloride-, barium chloride- and electrically induced arrhythmia rat models.

## 2. Results

Normal ECG waves with a sinus rhythm were observed in all investigated models before intravenous or subepicardial injection of test substances and before injection of arrhythmogens.

### 2.1. Calcium Chloride-Induced Arrhythmia

**Figure 1:**
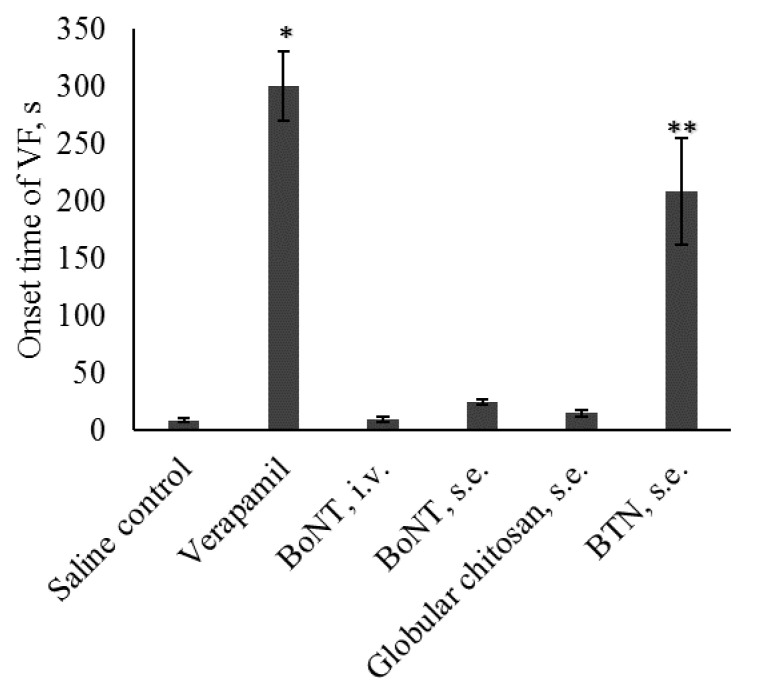
The initial onset time of ventricular fibrillation (VF) after injection of the different tested substances, in the calcium chloride model of arrhythmia. N = 10 for saline, BoNT (intravenously and subepicardially) and chitosan nanoparticle groups, *n* = 2 for Verapamil group and *n* = 5 for BTN group (see Table 1). * *p* < 0.01 vs. control, ** *p* < 0.01 vs. Verapamil, ANOVA with LSD post hoc test.

Intravenous injection of calcium chloride (150 mg/kg) caused severe lethal ventricular fibrillation (VF) after a few seconds p.i (Table 1, Figure 1). Neither BoNT/A1 intravenous or subepicardial injection nor subepicardial injection of chitosan nanoparticles prevented lethal VF. Subepicardial injection of BoNT/A1 lead to a slight, statistically insignificant increase in the onset time of VF (24.4 ± 2.1 s in the BoNT/A1 group vs. 8.2 ± 1.7 s in the control group, *p* = 0.288). Subepicardial BTN injection prevented lethal VF in five rats and led to a significant increase in the onset time of VF (208.6 ± 46.6 s in the BTN group vs. 8.2 ± 1.7 s in the control group, *p* < 0.001). However, verapamil was more effective than BTN and prevented lethal VF in eight animals, with an initial onset time of VF of 300.0 ± 30.0 s (*p* = 0.0000 vs. control, *p* = 0.002 vs. BTC group). Only rats demonstrating VF were included in the statistical analysis of the initial onset time of VF: there were 10 animals in the control, BoNT/A1 (i.v. and subepicardial) and chitosan nanoparticles groups, two animals in the verapamil group and five animals in the BTN group (Figure 1).

Due to all the tested substances, only BTN displayed a significant antiarrhythmic effect 15 min after subepicardial injection, and only this formulation was chosen for further study.

### 2.2. Barium Chloride-Induced Arrhythmia

Intravenous injection of barium chloride (7.5 mg/kg) caused premature ventricular contractions (PVC), followed by bigeminy 1–5 min after injection, followed by restoration of sinus rhythm. None of the animals perished.

In comparison with the saline control, subepicardial BTN injection significantly reduced the incidence of ventricular arrhythmias at doses between 0.5 and 5 U(BoNT/A1)/kg (Table 2). Unexpectedly, a 0.5 U(BoNT/A1)/kg dose proved more effective than 1 and 2 U/kg doses; however, these differences were not significant (*p* = 0.63 and 0.35, respectively).

### 2.3. Electrical Stimulation

**Figure 2:**
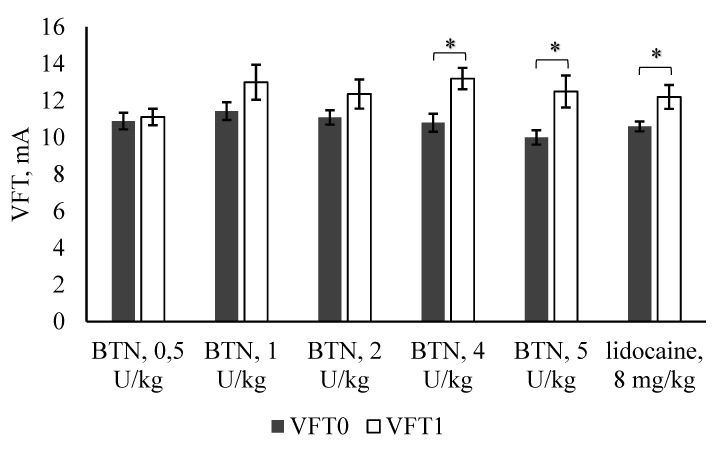
The effect of BTN (subepicardial injection) or lidocaine (i.v.) on VFT in anesthetized rats. VFT_0_—minimum electrical intensity that generated VF before injection of BTN or lidocaine; VFT_1_—minimum electrical intensity that produced VF after injection of BTN or lidocaine; mean ± SEM (* *p* < 0.05 VFT_1_ vs. VFT_0_; ANOVA with LSD post hoc test).

There were no statistically significant differences between the values of VFT_0_ (threshold of ventricular fibrillation before BTN or lidocaine injection) in different groups. Subepicardial BTN injection increased VFT_1_ (threshold of ventricular fibrillation after BTN or lidocaine injection) in a dose-dependent manner (Figure 2). VFT (ventricular fibrillation threshold) was increased by 12% and 10% upon administration of 1 and 2 U(BoNT/A1)/kg, respectively. These differences were not statistically significant. In contrast, VFT was significantly increased by 18% and 20%, upon administration of 4 and 5 U(BoNT/A1)/kg, respectively (*p* = 0.0136 and 0.0177, respectively). The reference antiarrhythmic lidocaine increased the VFT by 13% (*p* = 0.0344).

## 3. Discussion

Several studies have shown that temporary autonomic block using BoNT might be a novel therapeutic option for the treatment of postoperative AF [4,5,6,16]. It is well known that BoNT acts on neuromuscular junctions and blocks the exocytotic release of acetylcholine (ACh) stored in synaptic vesicles [17]. ACh is the main neurotransmitter of the parasympathetic nervous system and an internal transmitter of the sympathetic nervous system [18]. The role of the sympathetic and parasympathetic nervous system in the pathophysiology of cardiac arrhythmias is complex [19]. Selective ablation or stimulation of the different components of the autonomic nervous system, such as ganglionic plexi or the vagal nerve, can modulate the activity of this system and treat arrhythmias [20,21]. By blocking ACh release from the autonomic nerve terminals, BoNT can affect the parasympathetic control of the sinoatrial and atrioventricular node of the heart through the vagal nerve [3,4,22,23].

The antiarrhythmic effects of BoNT injection into ganglionated plexi have been shown to persist for at least one year after cardiac surgery [5,16]. However, it is important to find a way to enhance and further prolong this therapeutic effect. Indeed, patients developing new-onset postoperative atrial fibrillation have a high risk of recurrent atrial fibrillation for as long as two years after surgery [24,25]. Recently, we have demonstrated through intramuscular injection in rats [13] that globular chitosan prolongs the effect of BoNT/A1 and decreases its subsequent toxicity. The persistence of this effect on BoNT/A1 will be investigated in a future study. Here, we assessed the influence of globular (nanoprticle) chitosan on the antiarrhythmic properties of BoNT/A1.

Chitosan is a natural polymer known for its lack of toxicity and immunogenicity, its biodegradability and antimicrobial properties. As such, it is an excellent candidate for a variety of medical and pharmaceutical applications [26,27]. Thanks to their globular form and a high degree of diacylation and in contrast to linear chitosan, globular chitosan used previously [28] and the improved chitosan nanoparticles used in the present study (Novochizol) yield aqueous suspensions equivalent to bona fide solutions. This characteristic is essential for subepicardial or intravenous injection as a clinical application of this compound. As demonstrated in the chloride calcium-induced model of arrhythmia, chitosan nanoparticles alone did not display any antiarrhythmic or arrhythmogenic properties. Thus, the antiarrhythmic effect of BTN is due to the action of BoNT. Accordingly, the dose of BTN was measured as U(BoNT)/kg.

No good model of arrhythmia exists that brings together all the essential anatomopathological, electrophysiological, biochemical and molecular factors present in clinical practice [29]. In addition, the assessment of antiarrhythmic properties of BoNT has relied exclusively on experimental models of vagal stimulation [3,4,7] and rapid atrial pacing [6]. In the present work, we used calcium chloride, barium chloride and left ventricle electrical stimulation to devise three different experimental models of arrhythmia in rats. Intravenous infusion of calcium chloride induces ventricular arrhythmias in animals by increasing intracellular free calcium and opening calcium channels [30]. In contrast, barium chloride decreases outward potassium currents [31]. The influence of the drug on K and Na channels could be assessed in the model of electrical stimulation [32]. Protection against rhythm disturbances caused by these arrhythmogenic factors demonstrates the ability of a compound to act as a potential antiarrhythmic agent.

Intravenous injection is a conventional route of administration for antiarrhythmics used in clinics. Accordingly, we initially administered BoNT/A1 by intravenous injections. However, this mode of administration did not prevent the induced arrhythmias. Instead, antiarrhythmic effects (non-significant for BoNT and statistically significant for BTN) were observed when BoNT/A1 and BTN were injected subepicardially, 15 min before the injection of calcium chloride. One of the limitations in the chosen rat model is the quasi-impossibility to perform an injection in autonomic ganglia or fat pads or even in the wall of the left atrium. Indeed, although the density of small fibers and ganglia is the highest in the posterior part of the left atrium and around the antrum of the pulmonary veins [33,34], the rat’s heart is very small and the heart rate is very high. In addition, a dense network of Ach-containing nerves running over the epi- and endocardial surfaces of left and right ventricles and a widespread distribution of muscarinic ACh receptors throughout the ventricle have been demonstrated in different species [35,36,37,38,39,40,41]. Therefore, we injected the tested substances subepicardially, in the left ventricles.

Since a time-lapse is required for BoNT to block neuromuscular transmission [7,10,11], we injected BoNT/A1 or BTN 15 min before the injection of arrhythmogens. Our results in the calcium chloride model demonstrated that this delay was not sufficient for BoNT/A1 to show significant antiarrhythmic effects. In contrast, BTN demonstrated clear antiarrhythmic effects despite the short time-lapse between the injection of BTN and the injection of the arrhythmogen. At the same time, chitosan nanoparticles alone did not show antiarrhythmic effect. Therefore, we conclude that chitosan nanoparticles accelerate the effect of BoNT/A1. As the next step, we examined the dose-dependency of BTN effects on the incidence of arrhythmias in either a barium chloride model or after electrical myocardial stimulation of the left ventricle.

There were no significant differences between the antiarrhythmic effect at different doses of BTN (0.5–5 U(BoNT)/kg) in the barium chloride model. Instead, all dosages significantly prevented arrhythmias as compared to the control group. On the other hand, there was a clear dose-dependent antiarrhythmic effect of BTN in the electrically induced model of arrhythmia. In addition, in this model, the effects of the 4 and 5 U(BoNT)/kg doses were comparable to the effects of lidocaine (8 mg/kg).

Our results demonstrate that the chitosan nanoparticle formulation of BoNT/A1 prevents arrhythmia induced by an activation of Ca, K and Na channels. The mechanism of this effect remains unclear. Further study is needed to understand whether BTN acts on ionic channels directly or whether its effect is mediated by the influence on the autonomic nervous system.

In clinical practice, the use of BoNT or BTN for the treatment of postoperative atrial fibrillation may become a promising alternative to the radiofrequency ablation of ganglionated plexi. Ablation techniques cause permanent destruction of anatomic structures of the heart and may become proarrhythmic [42,43]. At the same time, postoperative arrhythmia has been shown to be a transient phenomenon that generally arises in the first week after an operation [44,45]. Accordingly, the temporary nature of the effect of BoNT and subsequent recovery of conduction of the autonomic nervous system constitute an advantage of the investigated technique. Another beneficial finding is that the injection of BoNT into the ganglionated plexi or a subepicardial injection do not cause permanent injury to the autonomic neurons and myocardium.

## 4. Materials and Methods

### 4.1. Test Substances

BoNT/A1 (Xeomin) was purchased from Merz Pharmaceutical Gmbh (Frankfurt am Main, Germany); each vial contained 100 U BoNT/A1.

Chitosan nanoparticles (Novochizol) were provided by Bosti Trading (Nicosia, Cyprus). The average molecular weight of the starting chitosan raw material (Chitoclear by Primex, Siglufjörður, Island) was 450–500 kDa, and the degree of deacetylation was at least 90%. While regular, linear chitosan is insoluble at physiological pH, so chitosan nanoparticles may be suspended in aqueous solutions and the resulting suspension may be assimilated to a solution.

BTN was formulated by dissolving the content of one vial (100 U of BoNT/A1) in 1 mL of a 0.25% suspension of chitosan nanoparticles in physiological saline. The formulation was used as early as after 24 h, and up to 10 days after preparation.

### 4.2. Animals

Male Wistar rats weighing 410 ± 40 g were provided by the vivarium of the Institute of Cytology and Genetics SB RAS (Novosibirsk, Russian Federation). The animals were housed in the vivarium of «E. Meshalkin National medical research center» of the Ministry of Health of the Russian Federation and were allowed free access to water and commercial laboratory complete food. Prior to the experiment, the animals had an acclimatization period of 14 days. A daily physical examination of the animals was performed in accordance with the regulatory requirements. Animals were blindly randomized into groups immediately prior to performing studies.

The use of animals in this study was approved by the Local Ethics Committee of «E. Meshalkin National medical research center» of the Ministry of Health of the Russian Federation. All parts of the protocol were performed in accordance with the recommendations for proper use and care of laboratory animals (European Communities Council Directive 86/609/CEE) and the principles of the Declaration of Helsinki.

### 4.3. Anesthesia

To induce anesthesia, rats were administered a subcutaneous injection of atropine (0.01 mg/kg) and were subsequently placed in an anesthesia induction chamber with a continuous supply of air containing sevoflurane (3–5%) (Gas Anesthesia System 21100, Ugo Basile, Gemonio, Italy and Small Animal Ventilator 683, Harvard Apparatus, Holliston, MA, USA). Subsequent to anesthesia, each animal was placed on the operating table, and a 24G peripheral intravenous catheter was inserted into the tail vein. Anesthesia was maintained using intravenous (i.v.) administration of 20 mg/kg sodium thiopental solution every 5–10 min. The same catheter was used for intravenous administration of other medications. Mechanical lung ventilation with indoor air was performed using a Rodent Ventilator device (Ugo Basile, Gemonio, Italy) via a tracheostomy tube with a diameter of 3 mm.

### 4.4. ECG Analysis

Invasive ECG monitoring was performed with peripheral electrode pads. A standard lead II ECG was recorded throughout the experiments using a Schiller AT-6 electrocardiograph (Schiller, Baar, Switzerland). An ECG recording rate of 50 mm/s was used.

The ventricular ectopic activity was assessed according to the diagnostic criteria advocated by Lambeth Conventions (II) [46]. The ECGs were analyzed to determine the onset of episodes of arrhythmias, including premature ventricular contraction (PVC), bigeminy, ventricular tachycardia (VT) and ventricular fibrillation (VF). VT was defined as PVCs lasting ≥4 beats. VF was defined as rapid, irregular QRS complexes.

### 4.5. Subepicardial Injections

To perform subepicardial injections, the rats were anesthetized as described above, and subsequently intubated and mechanically ventilated. To access the heart, median sternotomy was performed, with subsequent tissue fixation using fixation devices. The left lung was moved aside to expose the left ventricle, and the tested substances were injected subepicardially using an insulin syringe mounted with a 26G needle. ECG was monitored throughout the entire procedure. After 20 min of observation, the rats were euthanized by insufflation of an excessive volume of carbon dioxide for 15 min.

### 4.6. Assessment of the Antiarrhythmic Effect

The antiarrhythmic effect of the tested substances was assessed in three different models of arrhythmia (induction by calcium chloride, barium chloride or left ventricle electrical stimulation). The arrhythmogenic dose of calcium chloride and barium chloride was determined in a preliminary study as the smallest dose that induced heart rhythm disorders in 100% of the study animals. Clinically approved antiarrhythmics were used as controls for each model of arrhythmia.

### 4.7. Calcium Chloride-Induced Arrhythmia

Wistar rats were randomly divided into the following groups, comprising 10 animas each:

Group 1. Saline control (physiological saline, 0.9%);
Group 2. Verapamil, intravenously, 2 µg/kg;
Group 3. BoNT/A1, intravenously, 5 U/kg;
Group 4. BoNT/A1, subepicardially, 5 U/kg;
Group 5. Chitosan nanoparticles, subepicardially, 0.014 mg/kg;
Group 6. BTN, subepicardially, 5 U(BoNT/A1)/kg.

Arrhythmia was induced by the intravenous injection of 10% CaCl_2_ solution (Moschimpharmpreparaty, Moscow, Russian Federation) to reach a final dose of 150 mg/kg. The reference antiarrhythmic verapamil (Ozon, Samara, Russian Federation) was injected intravenously 5 min before the arrhythmogen. Each control rat received 0.1 mL saline 5 min before the injection of the arrhythmogen. Test substances (BoNT/A1, globular chitosan and BTN) were injected intravenously or subepicardially 15 min before the arrhythmogen.

### 4.8. Barium Chloride-Induced Arrhythmia

The following groups of animals, comprising 10 animas each, were investigated:

Group 1. Saline control (physiological saline, 0.9%);
Group 2. Amiodarone, 5 mg/kg;
Groups 3 to 7. Subepicardial injection of BTN (0.5, 1, 2, 4 and 5 U(BoNT/A1)/kg).

Arrhythmia was induced by an intravenous administration of 2% BaCl_2_ solution to reach a final dose of 7.5 mg/kg. The BaCl_2_ stock solution was prepared by dissolving 2 g of BaCl_2_ (Sigma-Aldrich, St. Louis, MO, USA) in 100 mL of physiological saline under aseptic conditions, and when no particulate matter was visible, the solution was sterilized by passing through a 0.1 μm syringe filter (Millipore, Burlington, MA, USA).

Amiodarone (Sanofi-Aventis, Paris, France) was used as a reference antiarrhythmic and was injected intravenously 5 min before BaCl_2_. Physiological saline was injected into the control rats 5 min before BaCl_2_. BTN was injected subepicardially 15 min before BaCl_2_.

### 4.9. Electrical Stimulation

Anesthetized rats were subjected to a thoracotomy and left ventricle electrical stimulation using two stainless steel stimulating electrodes. A pacing system analyzer (ERA 300, Biotronik, Lake Oswego, OR, USA) was used to deliver electrical rectangular impulses (pulse-width 5 m, frequency 16.6 Hz). Electrical intensity was initially set at 10 mA and increased in stepwise increments of 1 mA until VF was observed. This minimum electrical intensity that produced VF was set as the threshold current for induction of VF (VF threshold—VFT).

Six groups of animals were investigated, each group comprising 10 animals. VFT_0_ was recorded after thoracotomy but before BTN injection. Then, recovery of heart rhythm was observed for 10 min, followed by subepicardial injection of BTN or i.v. injection of lidocaine. VFT_1_ was recorded 15 min after injection of BTN (0.5, 1, 2, 4 or 5 U(BoNT/A1)/kg) or lidocaine (8 mg/kg).

### 4.10. Statistical Analysis

Statistical analyses were carried out using Statistica 13 (TIBCO Software, Palo Alto, CA, USA). Differences were considered significant when *p* < 0.05. The incidences of VF or arrhythmias (CaCl_2_ or BaCl_2_ models, respectively) were compared using two-tailed Fisher’s exact test.

The onset times of VF (calcium chloride model) and VFT (electrically induced arrhythmia) were expressed as mean ± standard error of the mean (SEM). ANOVA with LSD post hoc test was implemented to identify significant differences between groups.

## Figures and Tables

**Table 1:** The effects of test substances on CaCl_2_-induced arrhythmia incidence in anesthetized rats.

Test Substances	Sinus Rhythm	Lethal VF	PVC *, Bigeminy, Not VF	The Incidence of VF, *p* (vs. Saline Control, Fisher’s Exact Test)
Saline control	0	10	0	
Verapamil	8	2	0	<0.001
BoNT/A1, i.v.	0	10	0	
BoNT/A1, subepicardially	0	10	0	
globular chitosan, subepicardially	0	10	0	
BTN, subepicardially	3	5	2	<0.05

* PVC—premature ventricular contractions.

**Table 2:** The effects of test substances on arrhythmia incidence in anesthetized rats, BaCl2-induced arrhythmia.

Test Substances	Sinus Rhythm	Arrhythmia (PVC, Bigeminy)	*p* (vs. Saline Control, Fisher’s Exact Test, Two-Tailed)
Saline control	0	10	
amiodarone	10	0	<0.001
BTN, subepicardially			
0.5 U(BoNT/A1)/kg	8	2	<0.001
1 U(BoNT/A1)/kg	4	6	<0.05
2 U(BoNT/A1)/kg	5	5	<0.05
4 U(BoNT/A1)/kg	8	2	<0.001
5 U(BoNT/A1)/kg	8	2	<0.001
